# Proteomic profiling of kidney biopsies in nephrotic syndrome

**DOI:** 10.12688/wellcomeopenres.22633.1

**Published:** 2024-12-24

**Authors:** Emily Williams, Maryline Fresquet, Anna S Li, Craig Lawless, David Knight, Elizabeth Colby, Judy Watson, Gavin I Welsh, Moin A Saleem, Rachel Lennon

**Affiliations:** 1 Wellcome Centre for Cell-Matrix Research, The University of Manchester, Manchester, England, UK; 2 Biological Mass Spectrometry Cure Facility, The University of Manchester, Manchester, England, UK; 3 Bristol Renal, University of Bristol Medical School, Bristol, England, UK; 4 Department of Paediatric Nephrology, Royal Manchester Children’s Hospital, Manchester University Hospitals NHS Foundation Trust, Manchester Academic Health Science Centre, Manchester, England, UK

**Keywords:** NURTuRE cohort, nephrotic syndrome, minimal change, focal segmental glomerulosclerosis, laser-microdissection, global proteomics

## Abstract

**Background:**

Minimal change disease (MCD) and focal segmental glomerulosclerosis (FSGS) are patterns of kidney injury observed in the filtering units of the kidney known as glomeruli. These histological patterns are seen in kidney biopsies from individuals with idiopathic nephrotic syndrome (iNS), which occurs in both children and adults. However, there is some indication that MCD and FSGS are within the same phenotypic spectrum.

**Methods:**

From the NURTuRE cohort of individuals with NS, we performed laser microdissection and mass spectrometry analysis of kidney biopsy samples to identify proteomic patterns of disease. 56 individuals with iNS segregated by histological pattern (37 MCD and 19 FSGS) across three age groups: early childhood (0–6 years), late childhood (6–18 years) and adult (>18 years).

**Results:**

We found no distinct clustering of proteomic profiles between MCD and FSGS, but identified global differences in glomerular cell and extracellular matrix composition related to both histological pattern and age. The proteomic data are available via ProteomeXchange with identifier PXD053362.

**Conclusions:**

The lack of distinct clustering between MCD and FSGS in our study suggests shared biological processes between these injury patterns of iNS, supporting the hypothesis that they are part of the same disease spectrum. The global differences observed in glomerular cell and extracellular matrix composition suggest involvement of diverse biogeological processes as different patterns of iNS manifests in different age groups. This study also demonstrates the feasibility of pooling bioresources, central processing of heterogeneous tissue samples, and developing laser-microdissection and proteomic analysis methodology.

## Introduction

The NURTuRE biobank ^1^ is a resource for fundamental and translational research investigating the progression of kidney disease. This biorepository contains biological samples from over 3,000 individuals with chronic kidney disease (CKD) and 800 individuals with idiopathic nephrotic syndrome across the England, Wales, and Scotland ^2, 3^. These samples include DNA, plasma, urine and kidney biopsies therefore permitting deep molecular phenotyping.

Nephrotic syndrome (NS) is a clinical triad defined by massive proteinuria (>3.5g/24 hours), hypoalbuminemia and oedema ^4^. Under the age of 12, children with NS are generally treated empirically with high dose glucocorticoids without a kidney biopsy, which is reserved for those with atypical clinical features or with steroid resistant nephrotic syndrome (SRNS) ^5^. In older children and adults, NS is managed as distinct subtypes based on the histological diagnosis ^6^. Minimal change disease (MCD) and focal segmental glomerulosclerosis (FSGS) are the main histological patterns associated with NS affecting both children and adults. Compared with MCD, FSGS is associated with a higher likelihood of steroid resistance and progression to chronic kidney disease (CKD) ^6^. However, MCD and FSGS are histological patterns present in kidney biopsies and they do not differentiate the underlying aetiology of NS.

MCD and FSGS are characterised by diffuse foot process effacement and can both be caused by a podocyte injury event, such as circulating permeability factors causing podocyte dysfunction. It has been proposed that MCD and FSGS are manifestations of the same disease spectrum. While MCD represents early disease, FSGS develops as the disease progresses and relapses. In recurrent FSGS post-transplantation, early biopsy samples have been found to have no abnormalities on light microscopy but extensive podocyte foot process effacement on electron microscopy, as seen with MCD. It is likely that individual disease phenotypes result from the complex interactions between genetic predisposition, severity and duration of podocyte injury and other kidney disease risk factors such as hypertension ^7^. A new concept of autoimmune podocyte injury has emerged with the identification of circulating antibodies to the podocyte protein nephrin. These antibodies have been identified at high prevalence in children and adults with NS ^8, 9^. To further stratify patients, systems biology approaches are being employed to understand the molecular complexity of NS and ultimately to design targeted treatment options ^10, 11^. 

Although histological patterns in NS are well described, the proteomic composition across the spectrum from MCD to FSGS and between childhood and adult-onset diseases is poorly defined. We performed global proteomic analysis of laser-microdissected sections of kidney biopsies from patients with idiopathic NS collected as part of the NURTuRE biorepository. We aimed to define changes in both cell and extracellular matrix composition in the glomeruli and tubulointerstitium of kidney biopsy samples to identify components and pathways associated with age and histological pattern. We also explored the feasibility of incorporating proteomic analysis into clinical diagnostic pipelines, with samples collected from different kidney centres across the UK and analysed in a centralised core facility.

## Methods

### Human kidney samples from NURTuRE

Ethical approval was obtained to collect clinical data and use archival biopsy samples under the NephroS study, approved by South West - Central Bristol Research Ethics Committee (reference 09/H0106/72) on 16/12/2016. Written consent was obtained from patients (or their parents/guardians if under 18 years old) visiting nephrology centres within the UK. A mixed cohort of 56 children and adults with idiopathic NS with a kidney biopsy diagnosis of MCD or FSGS from 10 centres were included. Clinical presentation included primary steroid resistance, steroid sensitive disease, late onset steroid resistance, and NS associated syndromes (e.g. Nail Patella Syndrome and Denys-Drash Syndrome). Exclusion criteria included other causes of NS (IgA nephropathy, membranoproliferative glomerulonephritis, membranous nephropathy, vasculitis, systemic lupus erythematosus, diabetes, obesity, hypertension as reported in the NephroS Protocol v9 28-11-2019).

### Laser microdissection

Formalin fixed and paraffin embedded (FFPE) kidney biopsy blocks were retrieved from clinical recruitment sites ( Table 1). 10µm sections were cut and mounted on MMI membrane slides (Molecular Machines & Industries, #50103) and samples were stored for up to 6 months prior to further processing. Slides were dewaxed using the automated Leica ST5010XL system (programme: 2 cycles of xylene for 2min; 2 cycles of 100% ethanol for 2min then 1min; 1 cycle of 70% ethanol for 30s; 1 wash in water) . The slides were loaded onto the MMI CellCut Laser Microdissection microscope, and a slide scan was performed at 4x magnification to facilitate navigation across the biopsy. Under 20x magnification, the closed-shaped drawing tool was used to isolate up to 300,000µm ^2^ of glomeruli (excluding Bowman’s capsules), and the circle tool with set diameter 195.44µm was used to select 300,000µm ^2^ of tubulointerstitium (TI) per patient ^12^. Regions of interest were dissected using a laser focus setting of 420µm at 60% laser power and 80µm/sec velocity. Glomeruli and TI samples were collected using adhesive MMI transparent caps (MMI, #50204) and stored at 4°C for up to 1 week until all specimens were collected and prepared for mass spectrometry analysis. 

**Table 1.** Patient demographic details.

Sample ID	Age	Age group	Diagnosis	Sex	Site	Biopsy Date
14 49 17	2y 2m 3 y 7 m 1y 4m	0 to 6 0 to 6 0 to 6	FSGS FSGS FSGS	F F M	Bristol Bristol Leeds	8-7-2018 9-2-2018 12-5-2018
1 19 30	6y 1m 17y 11m 17y 1m	6 to 18 6 to 18 6 to 18	FSGS FSGS FSGS	F F F	Bristol Cardiff Cardiff	6-3-2008 5-1-2001 6-15-2018
15 20 39 41 2 23 46 50 28 36 8 5 7	37y 0m 52y 4m 46y 4m 29y 8m 66y 0m 46y 4m 44y 6m 52y 0m 36y 4m 28y 3m 21y 3m 40y 0m 25y 0m	18+ 8+ 18+ 18+ 18+ 18+ 18+ 18+ 18+ 18+ 18+ 18+ 18+	FSGS FSGS FSGS FSGS FSGS FSGS FSGS FSGS FSGS FSGS FSGS FSGS FSGS	F M F M M M M M F M F M F	Birmingham Adults Birmingham Adults Derby Derby Imperial Leeds Adults Leeds Adults Leicester Nottingham Nottingham Oxford Salford Salford	1-1-2018 6-28-1999 6-6-2014 5-12-2015 9-1-2017 7-16-2018 5-9-2019 4-30-2015 1-11-2016 3-26-2018 6-20-2016 1-1-2016 11-7-2010
34 42 54 4 25 22 32 16 29 37 21 31	2y 3m 2y 6m 1y 9m 2y 10m 2y 1m 3y 2m 2y 0m 4y 1m 5y 7m 3y 4m 3y 10m 2y 2m	0 to 6 0 to 6 0 to 6 0 to 6 0 to 6 0 to 6 0 to 6 0 to 6 0 to 6 0 to 6 0 to 6 0 to 6	MCD MCD MCD MCD MCD MCD MCD MCD MCD MCD MCD MCD	F F F M F M M M M M M F	Birmingham Childrens Birmingham Childrens Birmingham Childrens Bristol Leeds Manchester Manchester Nottingham Nottingham Nottingham Nottingham Childrens Nottingham Childrens	10-7-2014 1-5-2016 3-30-2019 12-28-2011 3-5-2015 2-25-2012 4-12-2006 9-2-2015 1-1-1988 6-15-2009 3-20-2012 11-25-2013
13 48 27 44 10 12 26 3 6	10y 0m 6y 0m 7y 1m 11y 0m 17y 2m 11y 6m 8y 0m 6y 4m 6y 1m	6 to 18 6 to 18 6 to 18 6 to 18 6 to 18 6 to 18 6 to 18 6 to 18 6 to 18	MCD MCD MCD MCD MCD MCD MCD MCD MCD	M F F M M M M M M	Birmingham Adults Birmingham Childrens Bristol Bristol Derby Leeds Manchester Salford York	1-1-1992 3-1-2011 2-20-2016 5-1-2018 1-1-2001 1-20-2016 3-13-2014 1-1-1987 12-12-1963
24 51 11 18 33 35 43 45 52 55 40 47 56 9 38 53	39y 0m 22y 9m 30y 2m 38y 7m 45y 0m 23y 4m 40y 9m 36y 0m 20y 0m 49y 4m 46y 0m 27y 5m 45y 5m 23y 11m 38y 1m 31y 7m	18+ 18+ 18+ 18+ 18+ 18+ 18+ 18+ 18+ 18+ 18+ 18+ 18+ 18+ 18+ 18+	MCD MCD MCD MCD MCD MCD MCD MCD MCD MCD MCD MCD MCD MCD MCD MCD	M M F F F M M M M F M F M M M F	Birmingham Adults Cardiff Derby Derby Derby Derby Derby Derby Derby Derby Nottingham Oxford Oxford Royal Free Royal Free Royal Free	1-1-1993 6-29-2019 10-8-2013 9-24-2015 9-12-2001 12-9-2014 06-10-2015 9-29-2017 2-17-2017 9-13-2016 3-5-2013 3-10-2010 12-12-2016 8-21-2015 2-9-2007 1-1-2000

### Sample preparation for mass spectrometry

Dissected tissue was resuspended in 25µl of lysis buffer (5% SDS, 50 mM TEAB pH 7.5) and transferred to a Covaris MicroTube AFA Fiber Pre-Slit Snap-Cap 6x16mm (Covaris, #520045) before ultrasonication using the following settings: duration 400 seconds, peak power 400, duty factor 40%, cycles/burst 50, average power 160 (Covaris LE220-plus focused ultrasonicator). Samples were reduced using 5mM dithiothreitol (DTT; Promega #P1171) for 10 minutes at 60°C, then alkylated with 15mM iodoacetamide (SLS, #I1149-5G) for 30 minutes (in the dark). The reaction was quenched using 5mM DTT and centrifuged at 14,000g for 10 minutes. The supernatant was acidified using a final concentration of 1.2% aqueous phosphoric acid (Merck, #79617). 165µl of S-Trap binding buffer (100mM TEAB pH7.1 in 90% methanol) was added to the 27.5µl volume of acidified protein lysate. The acidified methanolic lysate was then added to an S-trap column (Protifi, #C02-micro-80), and centrifuged at 4000g for 2 minutes. The captured proteins were washed 3 times with 150µl binding buffer before overnight trypsin digestion at 37°C. Trypsin (Promega, #V5113) was added to the top of the columns at a ratio of 1:10 wt:wt (enzyme:protein) in digestion buffer (50mM TEAB pH7.5). Proteins were eluted from the columns using three consecutive buffers; 65µl digestion buffer, centrifuged at 4000g for 2 minutes; 65µl 0.1% aqueous formic acid, centrifuged at 4000g for 2 minutes; and 30µl 0.1% formic acid in 30% acetonitrile, centrifuged at 4000g for 2 minutes. Samples were then stored at 4°C until the desalting step. 10µl of OLIGO™ R3 Reversed-phased resin (Thermo Scientific, #1133907) was added to each well of a 96-well filter plates and washed with 200µl 50% acetonitrile, centrifuged at 200 g for 1 minute, then washed twice with 200µl 0.1% formic acid in water. Samples were added to the wells, incubated for 5 minutes on a plate mixer, and centrifuged at 200 g for 1 minute. Peptides were washed with 200µl of 0.1% formic acid, mixed for 2 minutes, and centrifuged at 200 g for 1 min. Peptides were collected with two successive elution of 50µl of 0.1% formic acid in 30% acetonitrile. Samples werelyophilised by vacuum centrifugation and stored at 4°C until mass spectrometry analysis.

### Data acquisition

Peptides were analysed by liquid chromatography-tandem mass spectrometry (LC-MS/MS) using an UltiMate 3000 Rapid Separation LC system (Thermo Fisher) couple to an Orbitrap Exploris 480 mass spectrometer (Thermo Fisher). Mobile phase A was 0.1% formic acid in water and mobile phase B was 0.1% formic acid in acetonitrile, and the column used was a 75 µm x 250 mm, 1.7 µm CSH C18 analytical column (Waters). An injection volume of 4 µl was loaded into the loop and reverse flushed onto the analytical column at a flow rate of 300 nl/min. Sample separation was achieved using a multistage gradient over 85 minutes.

MS data was acquired in a data-dependent manner in positive mode. Full MS data was acquired over a scan range of 300 to 1750 Th using an automatic gain control (ACG) target of 300% and a max fill time of 25 mS at a resolution of 120,000. Fragmentation data was obtained from signals with a charge state of +2 or +3 and an intensity over 5,000. Fragmentation spectra were acquired with a resolution of 15,000 with a normalised collision energy of 30%, a normalised AGC target of 10%, first mass of 110 Th and a max fill time of 120 mS.

### Proteomic analysis and label free quantification

Raw data files were processed separately for glomerular and TI samples using MaxQuant software (v 1.6.10.43) (MaxQuant, RRID:SCR_014485) ^13^. Spectra were searched against the Human proteome obtained from Uniprot (November 2021) ^14^. Methionine oxidation and N-terminal acetylation were set as variable modifications, with carbamidomethylation of cysteine as a fixed modification. Precursor tolerance was set at 20ppm and 4.5ppm, for first and main search respectively, with MS/MS tolerance set at 20ppm and up to two missed cleavages were allowed. The false discovery rate (FDR) of peptide spectrum match (PSM) and protein were set at 0.01 and “Match between runs” was enabled. All statistical analyses were performed in R (v4.1.0) ^15^ using the MSqRob package (v0.7.7) ( https://www.Rproject.org/). Proteins were taken as significantly changing across conditions using a FDR threshold of 5%. The mass spectrometry proteomics data have been deposited to the ProteomeXchange Consortium via the PRIDE ^16^ partner repository with the dataset identifier PXD053362.

## Results

### Study design and experimental workflow

**Figure 1.**
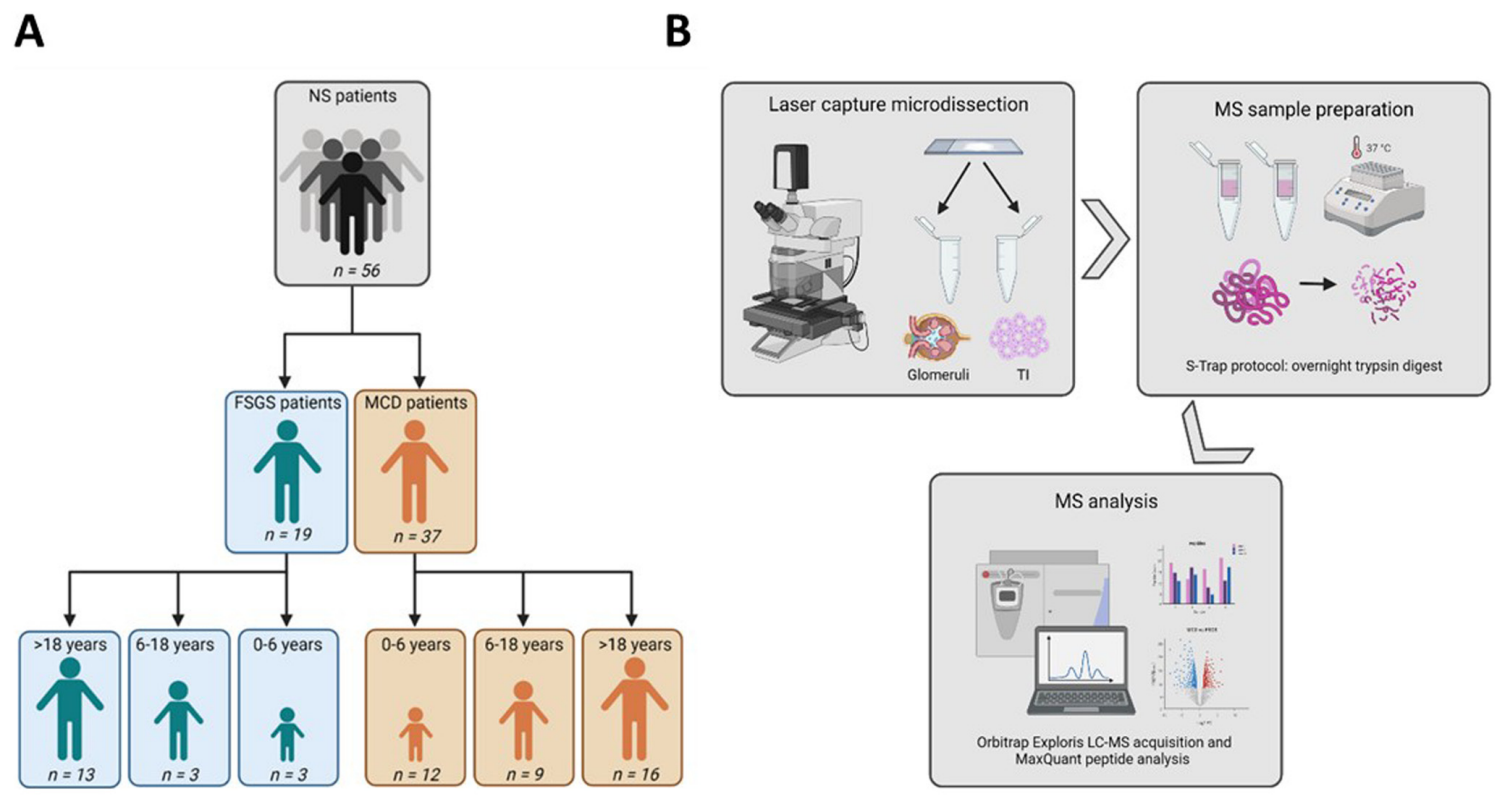
Study design and experimental workflow. **A**. Schematic of the study design (Patient grouping). **B**. Laser-microdissection /MS experimental workflow. Illustrations were created using BioRender software.

56 patients including 23 females and 30 males ( Table 1) with MCD (n=37) or FSGS (n=19) were subdivided into 3 age groups; 0–6, 6–18 and over 18 years old ( Figure 1A). Proportions of patients with idiopathic NS diagnosed with MCD decreased with age while those with FSGS increased. In the age 0–6 group, there were 9 patients with MCD and 3 with FSGS; the age 6–18 group consisted of 9 patients with MCD and 3 with FSGS; and the adult over age 18 groups consisted of 16 patients with MCD and 13 with FSGS. The biopsies were prepared for laser-microdissection as described in the experimental workflow ( Figure 1B). Briefly, glomeruli or tubulointerstitium (TI) were isolated from biopsy sections and captured using a laser-microdissection microscope. The collected glomerular and TI tissue samples were grouped according to histological pattern and age group.

### Identification of glomerular proteins from patient biopsies

**Figure 2.**
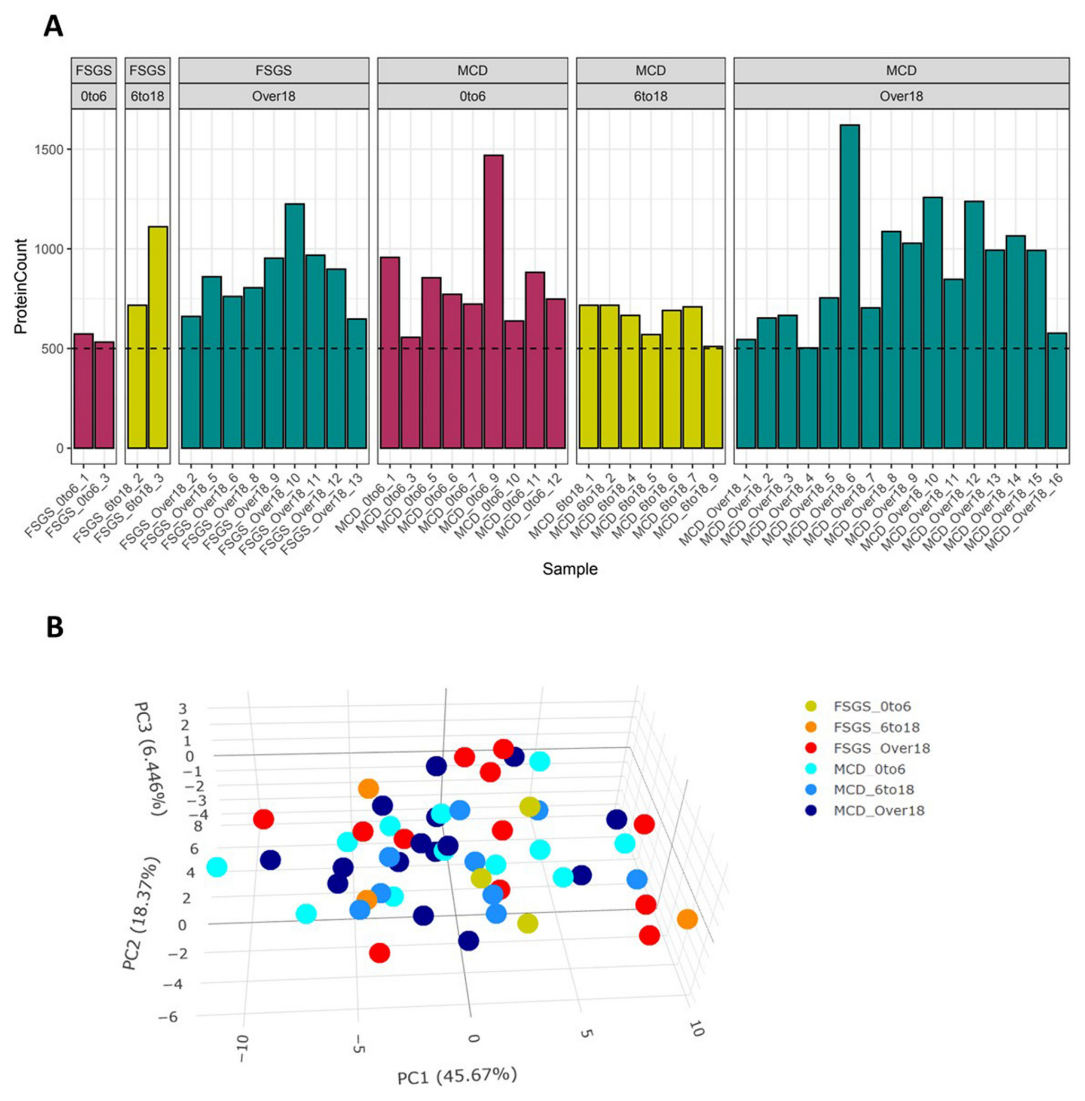
Glomerular proteins classified by histology and age group. **A**: Bar chart showing the number of proteins identified/quantified per sample with minimum of 500 proteins (2 outliers were excluded). **B**: PCA plot for samples grouped by disease condition and age range.

To define molecular patterns associated with MCD and FSGS, we isolated glomerular sections from all 56 NS biopsies, collecting between 13,000-300,000µm ^2^ of tissue (up to a maximum of 30 glomeruli). We identified 1729 proteins across all samples, and we proceeded to analyse samples containing at least 500 quantified proteins, leading to the exclusion of two samples. Whilst we observed some variation in the detection of proteins across the samples ( Figure 2A), a principal component analysis (PCA) based on quantitative MS data demonstrated no clear separation between samples grouped according to histological pattern or age. The lack of distinct clusters between or within each group suggests shared disease mechanisms, but it may also reflect the heterogeneity of the samples ( Figure 2B).

### Altered and extracellular matrix proteins in NS glomeruli

**Figure 3.**
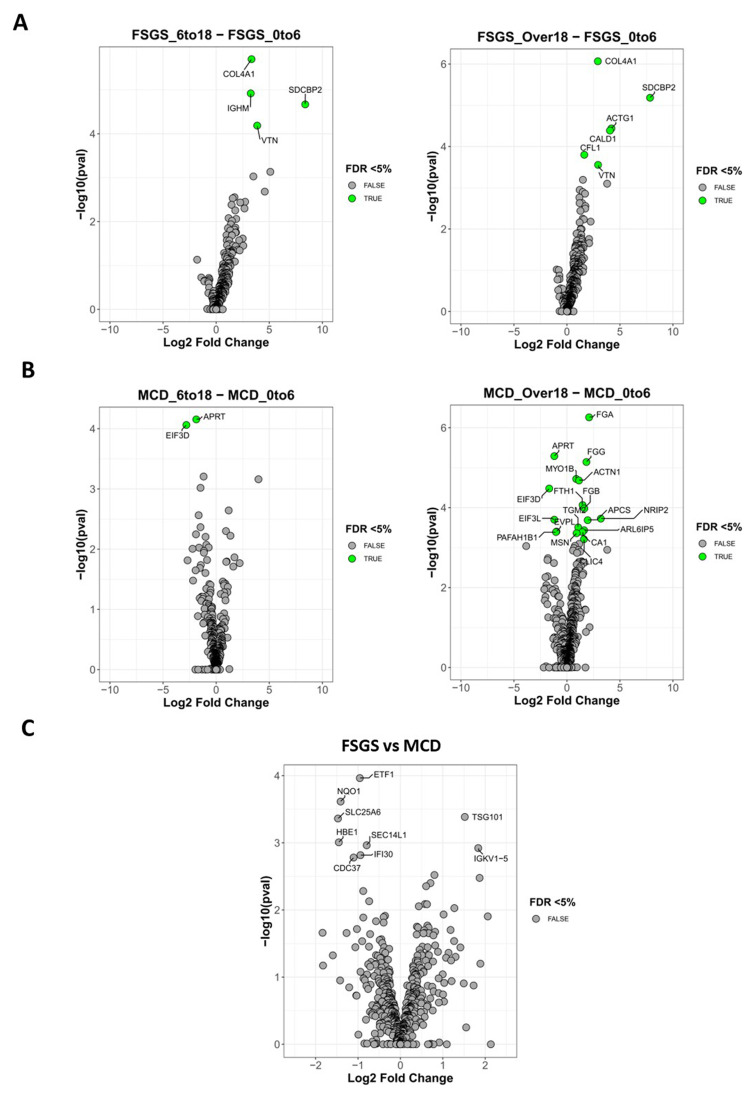
Altered glomerular proteins in nephrotic syndrome. Volcano plots of abundance levels of glomerular proteins; **A**: identified in FSGS across the different age groups. **B**: identified in MCD across the different age groups. **C**: Volcano plot of glomerular protein abundance levels comparing FSGS and MCD. Highlighted in green are the statistically significant higher-abundance or lower-abundance proteins with qval < 0.05.

Although the PCA did not identify distinct clusters, we did observe statistically significant differences in protein abundance in both FSGS and MCD groups over age ( Figure 3A–B). For both histological groups, we observed the biggest differences when comparing the adult groups to the early childhood group. In the adult FSGS group, CFL1, CALD1 and ACTG1 were all increased. These proteins are involved in actin binding and maintenance of the cytoskeleton. SDCBP2, which mediates cell signalling and the organisation of protein complexes was also increased in the adult FSGS group. In addition to these cellular components, we observed changes in extracellular matrix components. VTN and COL4A1 were both increased in the adult FSGS samples and may reflect increased age-related glomerulosclerosis. In the adult MCD group we also found a higher abundance of MYO1B, a class I myosin associated with the podocyte slit diaphragm and actin cytoskeleton ^17^, but most of the higher-abundance proteins in the adult MCD group were components of the immune system (MSN, FTH1, FGG, APCS, FGB, FGA and CA1) and involved in coagulation pathways (ACTN1 and fibrinogens). The lower-abundance proteins (EIF3D and EIF3L) are part of the translation machinery. This suggests immune-mediated pathways play a greater role in MCD development in the adult group. The analysis of FSGS versus MCD across all age groups revealed 6 lower abundance proteins (ETF1, NQO1, SLC25A6, HBE1, CDC37, IFI30, SEC14L1) and 2 higher abundance proteins (TSG101, IGKV1-5) ( Figure 3C). These proteins are involved in a diverse range of cellular functions, including translation regulation, redox regulation, mitochondrial function, lysosomal degradation, vesical trafficking and both innate and adaptive immunity. Overall, the differences in molecular profiles of isolated NS glomeruli suggests there is distinction between immune system involvement and extracellular matrix regulation according to age and histological pattern.

### Altered tubulointerstitial composition in NS

**Figure 4.**
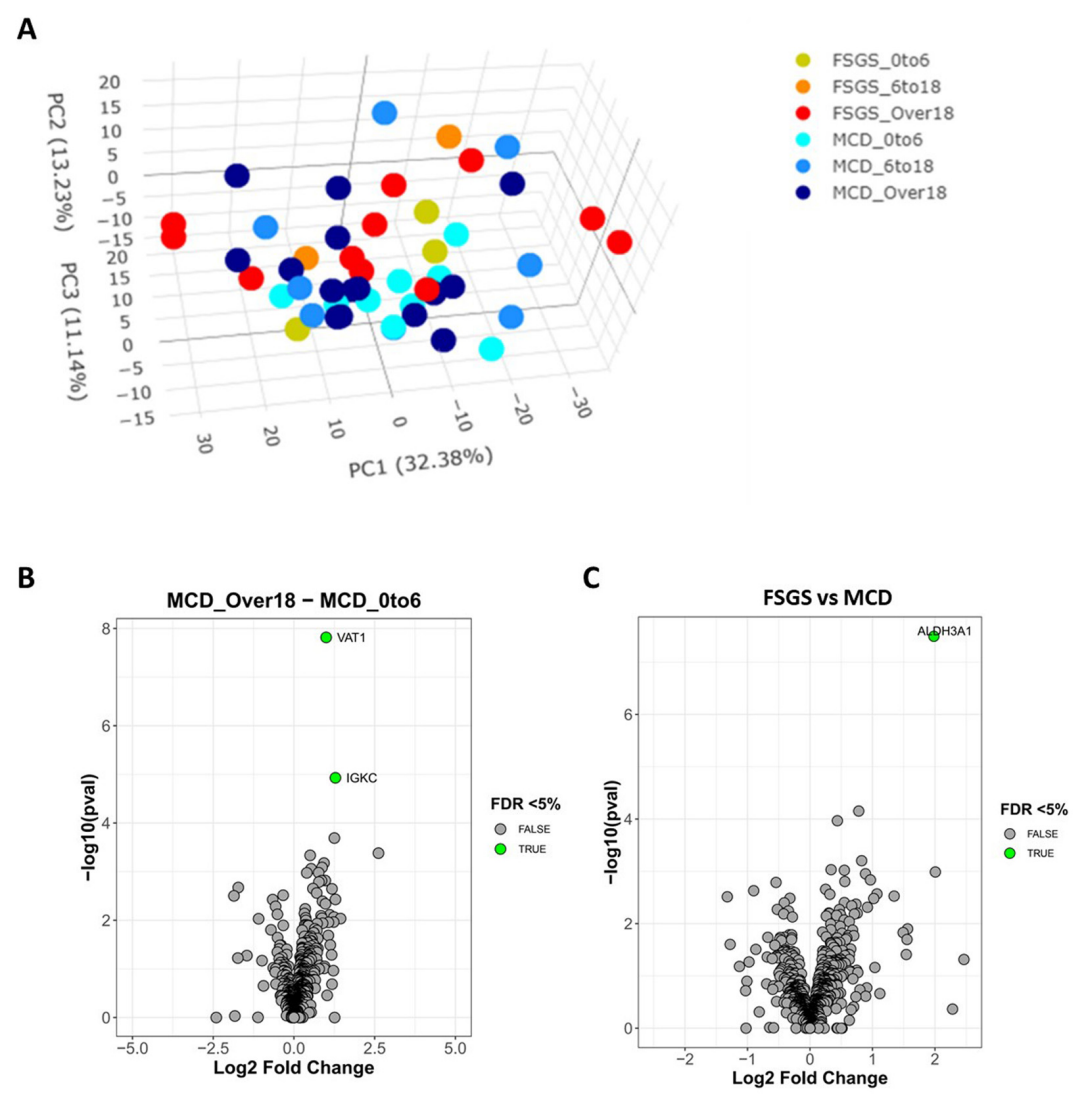
Altered tubulointerstitial proteins in nephrotic syndrome. **A**: 3D PCA plot for all samples. **B**: Volcano plot of tubulointerstitial protein abundance levels in MCD between age groups. **C**: Volcano plot of protein abundance levels comparing MCD and FSGS.

To determine changes in the tubulointerstitial (TI) compartment across the NS groups, we laser-captured 300,000µm ^2^ tissue from the same 56 NS patient biopsies. As with the glomerular analysis, a PCA showed no defined clusters between histological pattern and age group ( Figure 4A). This reflects the minimal interstitial fibrosis and tubular atrophy at the time of the biopsy. At the level of individual proteins and across all histology and age groups, we did not find significant changes in the TI data. Only two proteins were increased in the MCD group when comparing adults with the early childhood group and they were the vesicular transport protein VAT1 and the immunoglobulin protein IGKC ( Figure 4B). Between FSGS and MCD across all ages we found increased ALDH3A1, which is a protein involved in oxidative stress resistance ( Figure 4C). Once again this suggests greater involvement of the immune system in the adult MCD group and associations with altered protein trafficking and oxidative stress in NS.

## Discussion

With molecular profiling of NS patient biopsies, we discovered: 1) changes in both cell and extracellular matrix composition across age and histological pattern, and 2) differences in kidney compartments with more changes identified in the glomeruli than the tubulointerstitial tissue.

The lack of principal component analysis clustering suggests shared biological processes and lack of distinct disease mechanisms between FSGS and MCD across all age groups, supporting the hypothesis that FSGS and MCD are manifestation of the same disease spectrum. By comparing kidney biopsies in different age groups, we found age-related proteomic patterns between the MCD and FSGS disease groups. These observations suggest different underlying disease mechanisms in adult NS compared to young children. Most of the higher-abundance proteins were immune system components in the adult MCD group compared to the early childhood group, suggesting that immune-mediated mechanisms play a greater role in MCD development in adults. Indeed, the recent discovery of circulating antibodies to the slit diaphragm protein nephrin in children and adults with NS provides further evidence of the immune system driving pathology in NS ^8^. In the reported mouse model, anti-nephrin antibodies also induced MCD and glomerular proteomic analysis identified increased levels of MYO1B amongst other actin-binding or cytoskeletal proteins compared to untreated mice ^8^ **.** We also observed increased MYO1B levels in the adult MCD group compared to young children. MYO1B belongs to the Myosin 1 family of actin-associated molecular motors. Myosin 1 motors participate in a wide range of cellular processes related to generation and regulation of membrane tension ^18^. *In vitro* studies suggest that MYO1B acts as an actin depolymerase and could shape membranes by exerting force on actin filaments and regulating the rate of actin polymerisation ^19^. Although we observed fewer differences in protein abundance between the FSGS adult and early childhood groups, we found several proteins involved in actin-binding, maintenance of cytoskeleton and extracellular matrix, suggesting greater cytoskeletal and matrix alterations in the adult FSGS group.

The changes we observed in isolated glomeruli may represent glomerular responses to antibody injury. This may be different in adults who are generally pre-treatment with immunosuppression at the time of kidney biopsy, and a proportion of them are likely to be steroid-sensitive; whereas children generally have already received empirical treatment with corticosteroids and found to be steroid-resistant prior to the biopsy. Given also the wide age-range of the adult cohort and varied clinical features, these differences may also be related to underlying age-related glomerular changes and the heterogeneity within the patient cohort.

It is important to recognise the limitations of this study. The lack of principal component analysis clustering may also reflect the heterogeneity of the samples. This could be attributed to various factors such as sample collection and preparation. Samples were collected from 10 participating clinical sites and prepared using different protocols. For example, the duration for tissue fixation varied from centres to centres ( Table 2) which may impact the number of proteins detected across samples ^20^. In addition, the low sample volume of some glomerular samples may have also limited protein identification.

**Table 2.** Fixation times per sample collection site.

Centre	Fixation Time
Birmingham Adults	2–72 hours.
Cardiff	1 hour in warmed formalin, or 6–24 hours.
Derby	1 hour minimum.
Leeds	1–24 hours.
Manchester	4–5 hours.
Newcastle	6–24 hours.
Nottingham	1 hour minimum.
Portsmouth	15–70 hours.
Salford	24 hours
Sheffield	<24–72 hours.

Nevertheless, the study demonstrated feasibility of pooling bioresources, central processing of heterogenous tissues samples, and developing laser-microdissection and proteomic analysis methodology. A cohort of patients with well-defined phenotypes, greater tissue availability, and a strict biopsy fixation protocol would improve protein identification and help establish definitive associations between differences in protein abundance and disease phenotypes.

## Ethics and consent

Ethical approval was obtained to collect clinical data and use archival biopsy samples under the NephroS study, approved by South West - Central Bristol Research Ethics Committee (reference 09/H0106/72) on 16/12/2016. Written consent was obtained from patients (or their parents/guardians if under 18 years old) visiting nephrology centres within the UK.

## Data Availability

ProteomeXchange PRIDE database: Proteomic profiling of kidney biopsies in nephrotic syndrome. Accession number PXD053362; https://www.ebi.ac.uk/pride/archive/projects/PXD053362 ^21^.
