# Supplementary material for: Proteomic profiling of kidney biopsies in nephrotic syndrome
Source: Wellcome Open Res. 2024 Dec 24;9:731. [Version 1] doi: 10.12688/wellcomeopenres.22633.1 (PMC11845903; doi:10.12688/wellcomeopenres.22633.1)
Supplement: Peer review history

# Reviewer report: Reviewer response for version 1

**DOI:** 10.21956/wellcomeopenres.24934.r116554

**Published:** 2025-02-25

**Authors:** Federica Collino

**Affiliations:** 1 University of Milano, Clinical Sciences and Community Health, Milan, Italy

The manuscript entitled " *Proteomic Profiling of Kidney Biopsies in Nephrotic Syndrome"* is focused on the proteomic evaluation of kidney biopsy samples performed using laser microdissection and mass spectrometry analysis in 56 individuals with INS segregated by histological pattern and divided into three age groups.

**Strength and limitations**

**New innovative technology:** this paper confirms the feasibility of performing proteomics analysis using microdissection technology on biobank biopsies.

**Sample size and analysis:**

- An adequate sample size considering adult MCD and FSGS patient groups is present. The same was not true when early childhood (0–6 years) and late childhood (6–18 years) FSGS were considered. This limitation should be highlighted in the discussion. 
- Based on the data obtained on the influence of age on the same pathological state, it would be important also to run the comparison between MCD and FSGS separated by age group.
- The phrase in the results section's first paragraph is incorrect because patients with MDC in the representative cohort did not decrease with age. The phrase should be changed to "the proportion of INS diagnosed with FSGS increases with age...".
- Were biopsies collected at the disease appearance in all the patients? This should be defined as an inclusion criterion to permit a clear characterization of the patients at the same clinical time points.
- As reported by the authors, the sample dimension (recovered glomeruli) from each patient was variable and this could create bias by under- or over-represented glomeruli from specific patients. Based on this comment, the number of glomeruli or um2 per patient should be included.

**Discussion and citation:**

 - The implication of immune deregulation in adults and children is important and follows the data from the literature. Based on that, two observations can be made:

- The data on the presence of autoantibodies was reported mainly on the children’s population (the incidence of the anti-nephrin antibodies was lower in adults than in pediatrics). It would be important to comment on that.
- The incidence of nephrin antibodies was higher in steroid-sensitive patients in respect to steroid-resistant patients. The response to the immunosuppressive therapy needs to be indicated as it can provide also explanations for the proteomics data.

- The data generated from the tubular compartment should be commented in the discussion section.

Is the work clearly and accurately presented and does it cite the current literature?

Yes

If applicable, is the statistical analysis and its interpretation appropriate?

Yes

Are all the source data underlying the results available to ensure full reproducibility?

Yes

Is the study design appropriate and is the work technically sound?

Partly

Are the conclusions drawn adequately supported by the results?

Partly

Are sufficient details of methods and analysis provided to allow replication by others?

Yes

Reviewer Expertise:

Kidney pathophysiology, regenerative medicine, cellular biology

I confirm that I have read this submission and believe that I have an appropriate level of expertise to confirm that it is of an acceptable scientific standard, however I have significant reservations, as outlined above.

# Reviewer report: Reviewer response for version 1

**DOI:** 10.21956/wellcomeopenres.24934.r118273

**Published:** 2025-02-21

**Authors:** Robert W Hunter

**Affiliations:** 1 Royal Infirmary of Edinburgh, Queen’s Medical Research Institute, Edinburgh, UK

Thank you for inviting me to review this interesting paper.  

 This study addresses a very important question: do minimal change nephropathy and FSGS share a common molecular pathogenesis?  This is an important question because there is an unmet need for targeted therapies in these diseases, and understanding their molecular pathogenesis could help to develop new therapies.  

 The approach was to obtain glomerular and tubulointerstitial material from kidney biopsy samples by laser capture and then make an unbiased assessment of protein expression by mass spectrometry.  These are glomerular diseases, but decision to include tubulointerstium was a thoughtful strength.  The tubulointerstitium is affected by glomerular pathology so changes here  may shed light on disease pathogenesis.  Also, this compartment could be used to provide quality control reassurance of the experimental approach.  

 This is an intelligent way to address the question; the study was well designed and well conducted.  Given the relatively uncommon nature of these diseases, the patient numbers involved are impressively large.  (That said, there were still relatively few samples - an inherent problem with studying rare disease - but may impact on the interpretation of the results.)  As the authors point out, this work demonstrates the feasibility of conducting what must have been a technically demanding study.  It demonstrates the power and potential of well-curated biorespositories like NURTuRE.  

 So overall, this is an important, well-conducted and informative study.  I have some comments about some additional methodological details that would be helpful and how best to present and interpret the results.  I don't think there are any major flaws - these comments are questions about potential avenues that might improve the paper.  (In other words I don't think there is anything major changes that are essential - with the possible exception of clarifying what is being shown in Fig3C.)

**COMMENTS:**

**1) PATIENT DETAILS & DIAGNOSIS.  **It is not clear whether known monogenic causes of nephrotic syndrome were included / excluded / known.  Is it possible to include information (in aggregate, if not individual patient level) on the gene mutations - if known - within the different patient groups?  How was the histological diagnosis made?  Was this taken from the pathology report made during routine clinical care?  Is there any confirmation / measure of inter-observer variability?  (I don't think there would necessarily need to be - but useful to have this information in the paper.)

**2) DETAILS OF LASER CAPTURE. ** Presumably the regions sampled by laser capture might be subjective and / or operator dependent.  How did the authors approach this to minimise bias in sample selection?  For example, would it be possible that the operator was biased towards particular glomeruli based on morphology?  Were operators blinded to diagnosis?  How many glomeruli were obtained per sample: could those data be added to table 1?

**3) QC OF PROTEOMIC DATA.  **I am not very familiar with proteomic data, having never done this myself.  But presumably these datasets may be biased towards more abundant proteins - and therefore be enriched in certain protein classes (e.g. structural proteins)?  Is it helpful to present the number of different protein classes present in the dataset?  (Am not sure how these data are usually presented so am being vague in using the term "classes" - but perhaps categorising according to known biological function or cell compartment, analogous to a GO analysis?)  To give assurance that the proteomic data is high-quality, is it helpful to determine whether known glomerular proteins are high abundance in the glomerular sample and known tubular proteins abundant in the tubulointerstitial sample?  

**4) DECISION TO ANALYSE BY SUBGROUPS. ** Some of the subgroups, after stratification by both diagnosis and age, are very small (two patients per group for two of the groups).  This is likely to limit the power of the PCA analysis.  Does the PCA look different if only adult patients are included?  Also, why these subgroups?  Would it be interesting to compare male and female?  What about comparing different centres (the authors speculate in the discussion that different collection times may impact on the results - could this be tested in a PCA?)

**5) PRESENTATION OF PCA PLOTS.**  The 3D PCA plots are quite hard to interpret as they are presented projected onto a 2D plane.  Would the authors consider presenting - perhaps as supplemental - three plots in 2D (PCA1 vs. PCA2, PCA1 vs. PCA3, PCA2 vs PCA3)?  

**6) PRESENTATION OF VOLCANO PLOT IN Fig 3C.**  It is slightly unclear what the analysis presented in Fig3C showed.  There are 9 annotated proteins, implying that these were differentially regulated.  The main text states 8 differentially regulated proteins.  The figure legend states that none of these were significant (at FDR < 5%).  So how were these identified as being differentially regulated?  Also the direction of change is not immediately obvious in the figure (i.e. does upregulation mean higher in MCD or in FSGS)?

Is the work clearly and accurately presented and does it cite the current literature?

Yes

If applicable, is the statistical analysis and its interpretation appropriate?

Yes

Are all the source data underlying the results available to ensure full reproducibility?

Yes

Is the study design appropriate and is the work technically sound?

Yes

Are the conclusions drawn adequately supported by the results?

Partly

Are sufficient details of methods and analysis provided to allow replication by others?

Yes

Reviewer Expertise:

I am an academic nephrologist with expertise in studying the cellular and molecular mechanisms of renal disease in cell and animal models.

I confirm that I have read this submission and believe that I have an appropriate level of expertise to confirm that it is of an acceptable scientific standard.

# Reviewer report: Reviewer response for version 1

**DOI:** 10.21956/wellcomeopenres.24934.r116557

**Published:** 2025-01-13

**Authors:** Manoj Anant Dhanorkar

**Affiliations:** 1 Nephrology, All India Institute of Medical Sciences, Nagpur, Nagpur, Maharashtra, India

- The work done in this study is well thought out and planned however the selection of the sample from the repository with limited information is something that needs evaluation. 
- MCD and FSGS biopsy proven patients could have been selected and followed up to ascertain the nature of their disease as not all MCD progress to FSGS. The vary nature of steroid resistance in either of the two diseases should have been available to illustrate the similarity or difference of the selected population.
- So, for the final interpretation of the results, further details regarding the patients selected and the natural course of the illness, treatment details is imperative.
- I suppose the data is reproducible as the methodology is very well explained and available for evaluation in full detail.
- The final conclusion drawn is still not completely acceptable as previous urinary proteomics have shown difference between MCD and FSGS.(1)

Is the work clearly and accurately presented and does it cite the current literature?

Yes

If applicable, is the statistical analysis and its interpretation appropriate?

Partly

Are all the source data underlying the results available to ensure full reproducibility?

Yes

Is the study design appropriate and is the work technically sound?

Partly

Are the conclusions drawn adequately supported by the results?

Partly

Are sufficient details of methods and analysis provided to allow replication by others?

Yes

Reviewer Expertise:

Nephrology, Renal glomerular disease, Genomics

I confirm that I have read this submission and believe that I have an appropriate level of expertise to confirm that it is of an acceptable scientific standard, however I have significant reservations, as outlined above.

## References

[1] : Comparative differential proteomic analysis of minimal change disease and focal segmental glomerulosclerosis. https://doi.org/10.1186/s12882-017-0452-6 10.1186/s12882-017-0452-6 PMC529195728158993

# Response

**Published:** 2025-02-03

**Authors:** Anna Shanshan Li

**Affiliations:** Wellcome Centre for Cell-Matrix Research, The University of Manchester, Manchester, England, UK

Thank you for taking time to review our manuscript. We have the following responses to the points you have raised.

**Limitation of the study** Sample Selection: We acknowledge that the information available for sample selection from the NURTuRE repository was limited.  However, this limitation is inherent in utilising a pre-existing biorepository.  We have stated this limitation in the Discussion section of our manuscript.   

 Clinical Data: Our focus for this paper is to report the proteomic profiles of these biopsies, which provide valuable insights into the molecular landscape of MCD and FSGS. We acknowledge that further details regarding the patients and their clinical course would be valuable. However, as mentioned in the manuscript, the clinical information available in the NURTuRE repository was limited. We have also highlighted this limitation in the Discussion section.   

**MCD and FSGS in nephrotic syndrome** Our findings support the hypothesis that MCD and FSGS may represent different manifestations of the same pathology spectrum.  However, this does not necessarily imply that MCD is an earlier stage that inevitably progresses to FSGS.    Steroid Resistance: As discussed in our manuscript, there is a difference in the timing of biopsies between children and adults, with children typically undergoing a trial of steroid treatment before a biopsy is considered, while adults tend to be biopsied at the time of disease presentation.  It is important to note that steroid resistance is not a reliable distinguishing factor between MCD and FSGS, and this has already been reported in the literature.

**Tissue Proteomics vs. Urine Proteomics:** It is important to emphasise that tissue proteomics and urine proteomics provide distinct information.  Tissue proteomics reflects the protein composition within an organ itself, while urine proteomics captures proteins from multiple tissue origins.  Therefore, it is not surprising that our findings may differ from previous studies that have focused on urine proteomics.   

**Conclusion:** We believe that our conclusions are adequately supported by the results of our study.  We have identified both significant overlaps and differences in protein expression between MCD and FSGS across different age groups.  These findings provide new insights into the molecular landscape of these histological patterns of nephrotic syndrome.

# Reviewer report: Reviewer response for version 1

**DOI:** 10.21956/wellcomeopenres.24934.r116551

**Published:** 2025-01-13

**Authors:** Andrea Angeletti

**Affiliations:** 1 Dialysis and Transplantation Unit, IRCCS Istituto Giannina Gaslini, Genoa, Italy

**Summary of the Article**

 The manuscript titled *Proteomic Profiling of Kidney Biopsies in Nephrotic Syndrome* is a comprehensive study leveraging proteomic techniques to explore molecular differences in kidney biopsies from patients with idiopathic nephrotic syndrome (iNS). The authors focus on Minimal Change Disease (MCD) and Focal Segmental Glomerulosclerosis (FSGS), employing laser microdissection and mass spectrometry to analyze kidney samples across three age groups.

 Key findings include:

- Lack of distinct clustering between MCD and FSGS, suggesting a shared biological mechanism.
- Identification of age-related differences in glomerular proteins, particularly in immune and cytoskeletal components.
- Validation of the feasibility of integrating proteomics into diagnostic workflows.

This study adds valuable insights into the molecular landscape of nephrotic syndrome and supports the hypothesis of a disease spectrum between MCD and FSGS.

**Strengths**

- **Innovative Methodology**: The use of laser microdissection combined with proteomics is cutting-edge and provides high-resolution molecular insights.
- **Comprehensive Analysis**: The study includes patients across diverse age groups, offering a unique perspective on disease progression.
- **Data Accessibility**: The availability of proteomic data in public repositories (Proteome change) enhances transparency and reproducibility.
- **Translational Potential**: The study demonstrates the feasibility of incorporating advanced proteomics into clinical diagnostics.

**Points to Address**

 To strengthen the manuscript and ensure scientific rigor, the following issues should be addressed:

1. **Sample Heterogeneity**:
  - The lack of clustering in the PCA may partly reflect the heterogeneity in sample collection and processing across 10 centers. This limitation should be discussed in greater detail, with specific recommendations for standardizing protocols in future studies.
2. **Small Sample Size for Certain Subgroups**:
  - Some age groups (e.g., early childhood) have limited samples for robust statistical analysis. While acknowledged, this could be mitigated by pooling additional data from similar cohorts or conducting meta-analyses.
3. **Clinical Relevance**:
  - While the proteomic differences are highlighted, the implications for patient stratification or targeted therapy remain underexplored. Suggestions for how these findings could guide treatment decisions should be elaborated.
4. **Comparison with Existing Literature**:
  - The discussion could benefit from a deeper comparison with other recent proteomic studies in nephrotic syndrome, highlighting similarities or novel contributions.
5. **Discussion of Negative Results**:
  - The absence of significant findings in the tubulointerstitial compartment warrants a brief discussion to clarify whether this reflects biological phenomena or technical limitations.

**Conclusion**

 This manuscript represents a significant contribution to the understanding of nephrotic syndrome through proteomics. I strongly recommend this article for indexing following minor revisions to ensure scientific soundness and maximize its translational value.

Is the work clearly and accurately presented and does it cite the current literature?

Yes

If applicable, is the statistical analysis and its interpretation appropriate?

Yes

Are all the source data underlying the results available to ensure full reproducibility?

Yes

Is the study design appropriate and is the work technically sound?

Yes

Are the conclusions drawn adequately supported by the results?

Yes

Are sufficient details of methods and analysis provided to allow replication by others?

Yes

Reviewer Expertise:

Immunology

I confirm that I have read this submission and believe that I have an appropriate level of expertise to confirm that it is of an acceptable scientific standard.

# Response

**Published:** 2025-02-03

**Authors:** Anna Shanshan Li

**Affiliations:** Wellcome Centre for Cell-Matrix Research, The University of Manchester, Manchester, England, UK

We thank the reviewer for their positive evaluation of our manuscript and insightful comments. We especially appreciate the recognition of our innovative methodology and the translational potential of this work, which highlights the impact our approach can have on the field of nephrology. We acknowledge the potential for sample heterogeneity introduced by variations in collection and processing across the different centres. This is an inherent challenge when working with a valuable resource like the NURTURE biorepository. While we could not retrospectively control for these variations, we have transparently identified this as a limitation and advocate for the implementation of standardised protocols in future studies. We also agree that the small sample size, especially in certain subgroups, might limit the broader applicability of our findings. Nonetheless, we believe our study serves as an important proof-of-concept, demonstrating the feasibility and value of applying proteomic analysis to kidney biopsies. These findings pave the way for larger, multi-centre studies with greater statistical power. Due to these limitations, we have been cautious about drawing extensive conclusions or delving too deeply into clinical relevance and negative results. However, we believe this study provides a valuable foundation for future research in this area and are excited about the potential of proteomics to improve our understanding and treatment of nephrotic syndrome. The establishment of a new study with standardised protocols will undoubtedly help overcome the current limitations and contribute to a deeper understanding of the molecular mechanisms underlying nephrotic syndrome.
